# Molecular Epidemiology and Risk Factors of Carbapenem-Resistant *Klebsiella pneumoniae* Infections in Eastern China

**DOI:** 10.3389/fmicb.2017.01061

**Published:** 2017-06-13

**Authors:** Bing Zheng, Yingxin Dai, Yang Liu, Weiyang Shi, Erkuan Dai, Yichao Han, Dandan Zheng, Yuetian Yu, Min Li

**Affiliations:** ^1^Department of Laboratory Medicine, Renji Hospital, School of Medicine, Shanghai Jiao Tong UniversityShanghai, China; ^2^Department of Critical Care Medicine, Renji Hospital, School of Medicine, Shanghai Jiao Tong UniversityShanghai, China

**Keywords:** carbapenem-resistant, *Klebsiella pneumoniae*, risk factors, epidemiology, PFGE

## Abstract

**Background:** The increasing prevalence of carbapenem-resistant *Klebsiella pneumoniae* (CRKP) poses an immediate threat to treatment worldwide. This retrospective study assessed the molecular epidemiology and determined the risk factors for and outcomes of CRKP infections in a general teaching hospital in Shanghai, China.

**Methods:** From January 2013 to July 2015, 100 consecutive unique CRKP isolates isolated from hospitalized patients were collected. Isolates were screened for antibiotic resistance genes by polymerase chain reaction and molecular typing was performed by pulsed-field gel electrophoresis (PFGE). Patients infected with CRKP comprised the case group and were compared to the control group of patients infected with carbapenem-susceptible *Klebsiella pneumoniae*. Therapeutic effects were compared in the CRKP infection group.

**Results:** Among the 100 CRKP isolates, the percentages of multidrug-resistant, extensively drug-resistant (XDR), and pandrug-resistant were 50.0, 50.0, and 0%, respectively. All the CRKP isolates produced KPC-2 and could be divided into 18 PFGE clusters (A–O) and 70 subtypes. No dominant intra-hospital PFGE type was detected using a cutoff of 80% similarity. The ratio of CRKP infection to colonization was 51 to 49. Risk factors correlated with CRKP infection included pulmonary disease (*p* = 0.038), ICU stay (*p* = 0.002), invasive ventilation (*p* = 0.009), blood transfusion (*p* = 0.028), parenteral nutrition (*p* = 0.004), sputum suction (*p* = 0.006), medical history of previous hospitalization (*p* = 0.022), exposure to antibiotics 90 days before infection (*p* = 0.030), and antibiotic exposure during hospital stay including carbapenems (*p* = 0.013), enzyme inhibitors (*p* = 0.021), nitroimidazoles (*p* = 0.029), and glycopeptides (*p* = 0.000). Multivariable analysis showed that sputum suction (odds ratio 3.090, 95% confidence intervals 1.004–9.518, *p* = 0.049) was an independent risk factor for CRKP infections. Patients infected with CRKP with longer carbapenems treatment course (*p* = 0.002) showed better outcome.

**Conclusion:** This study showed the severity of CRKP infection in eastern China. Sputum suction was an independent risk factor for CRKP infection. Prolonged duration of treatment with carbapenems benefited the patients infected with CRKP.

## Introduction

*Klebsiella pneumoniae* (KP), one of the most commonly isolated Gram-negative bacterial pathogens in nosocomial infections, has posed a major threat to clinical and public health because of its ability to produce carbapenemases, which hydrolyze carbapenem antibiotics. Carbapenems constitute the last line of defense against infections caused by multidrug-resistant (MDR) Gram-negative organisms. The first case of carbapenem-resistant *Klebsiella pneumoniae* (CRKP) was reported in MacKenzie et al. (1997), after which it spread worldwide and outbreaks were reported in several countries (Meybeck et al., 2014; Zweigner et al., 2014; Matsumura et al., 2015; Stillwell et al., 2015). In China, the prevalence of CRPK has rapidly increased from 2.9% in 2005 to 13.4% in 2014 (Hu F.-P. et al., 2016) and the distribution differed greatly by region, with the lowest prevalence in the northeast and the highest prevalence in the eastern region of China (Xu et al., 2016).

Carbapenem-resistant *Klebsiella pneumoniae* infections have a poor prognosis, with a mortality rate of approximately 40–50% (Borer et al., 2009; Hoxha et al., 2016). Inferior outcomes are especially significant in high-risk immunocompromised patients including those with solid organ transplants, in intensive care units, and hematological malignancies (Kalpoe et al., 2012; Satlin et al., 2014; Simkins et al., 2014; Vardakas et al., 2015). Bloodstream infections caused by CRKP increase the risks of treatment failure and death (Tumbarello et al., 2012). As colistin is one of the few available antibiotics against CRKP, the therapeutic options for infections are limited. However, with the appearance of colistin resistant strains, treatment of CRKP infections is increasingly difficult (Capone et al., 2013).

Resistance to carbapenems is associated with several mechanisms. Carbapenemases, which are largely responsible for resistance, are classified into three functional groups: class A serine carbapenemases (for example, KPC), class B metallo-β-lactamases (for example, NDM), and class D β-lactamases (for example, OXA) (Bush, 2013). Among the above types of carbapenemases, KPC-producing KP has spread widely throughout the world in the past few years. Other factors contribute less to resistance, including the production of β-lactamases with weak carbapenemase activity, especially class A extended-spectrum β-lactamases (ESBLs) and class C AmpC lactamases, permeability defects and loss of porins (Ompk35 and Ompk36) (Tsai et al., 2013; Pitout et al., 2015).

Recently, outbreaks of CRKP have become increasingly common in China (Wang et al., 2014; Zhou et al., 2015) and have been recognized as a tremendous challenge. High mortality rates and lack of effective treatments place the patients into a perilous situation after infection with CRKP. Identification of risk factors for CRKP infection would improve the choice and efficacy of empirical therapy (Kofteridis et al., 2014), which could assist in early recognition and timely intervention. In addition, the majority of studies on the risk factors of CRKP are carried out in the United States, where the dominant clone of KPC-producing KP is ST256, in contrast to China, where the dominant strain is ST11 (Qi et al., 2011). Our study comprehensively described 100 CRKP clinical isolates collected in a tertiary teaching hospital in Shanghai, China, between January 2013 and July 2015. The study examined three dimensions: antibiotic resistance genes, molecular genotypes, and patient risk factors.

## Materials and Methods

### Study Design and Bacterial Isolates

The retrospective observational study was conducted in a comprehensive teaching hospital in Shanghai, China (Renji Hospital affiliated to Jiaotong University). This is a centrally located large teaching hospital in Shanghai (1,600 beds) with approximately 8,000 admissions/day. Consecutive non-duplicate strains from patients infected and/or colonized by CRKP between January 2013 and July 2015 were stored at -80°C and included in this study. All the CRKP isolates were identified by matrix-assisted laser desorption ionization time-of-flight mass spectrometry (MALDI-TOF MS, Bruker Daltonics, Bremen, Germany).

To study the risk factors of patients infected by CRKP infection, a stepwise matching technique was used to identify appropriate control cases from patients infected with carbapenem-susceptible *Klebsiella pneumoniae* (CSKP). Controls were matched to cases who were in the same ward during the same period (within 30 days) and were within 5 years of their age. If several patients in the control group met the qualification, one was randomly selected. An independent researcher, blinded to the objectives of the study, selected the control group according to these inclusion criteria. Informed consent was waived by The Institutional Review Board of Renji Hospital as all the patients’ personal information was de-identified and was unknown to the authors, and the collection of isolates was also approved.

### Antibiotic Susceptibility Test

The susceptibility of CRKP isolates to 18 antibiotics, including amikacin (AK), penicillin (P), ampicillin (AMP), ampicillin/sulbactam (SAM), piperacillin (PIR), cefazolin (CFZ), cefaclor (CEC), cefuroxime (CFM), cefotaxime (CTX), ceftazidime (CAZ), ciprofloxacin (CIP), trimethoprim-sulfamethoxazole (SXT), fosfomycin (FOS), cefoperazone/sulbactam (SCF), cefepime (FEP), cefoxitin (FOX), aztreonam (ATM) and minocyline (MH), was determined by Kirby-Bauer disk diffusion test (Oxoid, United Kingdom). Five antibiotics, including imipenem (IMP), meropenem (MEM), ertapenem (ETP), tigecycline (TGC) and polymyxin B (PB), were tested by microbroth dilution test following the criteria of the Clinical and Laboratory Standards Institute (CLSI) (CLSI, 2016). The minimal inhibitory concentrations (MICs) breakpoint for tigecycline was defined according to the European Committee on Antimicrobial Susceptibility Testing (EUCAST) (EUCAST, 2017), while the others were interpreted according to CLSI protocols (CLSI, 2016). *K. pneumoniae* ATCC700603 and *Escherichia coli* ATCC25922 were used as quality control strains for the antibiotic susceptibility tests.

### Identification of Antibiotic Resistance Genes

Crude DNA extracts were prepared by boiling the bacterial suspensions (Clark et al., 1993). This DNA was used as template in polymerase chain reactions (PCR) to detect antibiotic resistance genes. Standard PCR conditions were used to amplify genes encoding carbapenemases (*bla*KPC-2, *bla*OXA-48, *bla*VIM-1, *bla*VIM-2, *bla*IMP-1, *bla*IMP-2, and *bla*NDM-1), ESBLs (*bla*TEM, *bla*SHV, *bla*CTX-M-1, *bla*CTX-M-9, *bla*CTX-M-15, and *bla*CTX-M-25), plasmid-mediated AmpC (*bla*EBC, *bla*DHAM, *bla*CIT, *bla*FOX, *bla*ACC, and *bla*MOX), and quinolone resistance determinants (*qnr*A, *qnr*B, and *qnr*S) using primers and PCR conditions described previously (Carlone et al., 1986; Joyce et al., 1992; Shibata et al., 2003; Smith Moland et al., 2003; Poirel et al., 2004; Gay et al., 2006; Kruttgen et al., 2011; Doumith et al., 2012). The *bla*KPC-2 gene was the predominant carbapenemase gene found in *K. pneumoniae* in China (Hu L. et al., 2016). The PCR amplicons of *bla*KPC-2 and *bla*CTX-M were sequenced and the sequences were analyzed on the National Center for Biotechnology Information (NCBI) website^1^.

### Pulsed-Field Gel Electrophoresis (PFGE)

Molecular typing was performed by pulsed-field gel electrophoresis (PFGE). Genomic DNA was fragmented by restriction endonuclease ApaI (TaKaRa, Tokyo, Japan), and the DNA fragments were separated by electrophoresis on 1% SeaKem Gold agarose (Lonza, Rockland, ME, United States) in 0.5× TBE (45 mM Tris, 45 mM boric acid, 1.0 mM EDTA; pH 8.0) buffer using the CHEF Mapper XA PFGE system (Bio-Rad, Unietd States) at 6 V/cm and 14°C, with alternating pulses at a 120° angle in a 5–20 s pulse time gradient for 19 h. The PFGE results were visually assessed, and a dendrogram was generated by unweighted pair group method using arithmetic average (UPGMA) clustering in NTSYS software version 2.1 (State University of New York, Stony Brook, NY, United States). The isolates were placed into one PFGE type (i.e., clonally related) if the Dice coefficient was >80% (correlation coefficient > 0.80) (Endimiani et al., 2009; Chiu et al., 2013; Cheng et al., 2016), while patterns with no differences in fragments were considered to be of the same subtype (Tenover et al., 1995).

### Data Collection

After patients in both the case and control groups had been confirmed, data were collected by two trained reviewers from the electronic medical records and clinical microbiology laboratory databases. Thirty-nine clinical variables were applied in the analysis of the risk factors and evaluated for an interval of 90 days prior to the occurrence of KP infection. These variables included (1) demographics (gender, age); (2) baseline diseases (hypertension, diabetes mellitus, heart disease, malignancy, chronic pulmonary disease, liver disease, hematologic disease, nervous system disease); (3) community or nosocomial acquired infection; (4) previous therapeutic processes during the hospital stay, including length of hospital stay prior to KP infection, immunosuppressant therapy (>14 days of corticoid drugs or other immunosuppressants, or receipt of >1 dose of chemotherapy or immunosuppressants), ICU stay, surgery, blood transfusion, invasive device use (invasive ventilation, parenteral nutrition, drainage tube, urinary catheter, vascular catheter, sputum suction, nasogastric feeding tube); (5) medical history (90 days before KP infection), including previous hospitalization and exposure to antibiotics; (6) antibiotic exposure during hospital stay (cephalosporins, carbapenems, quinolones, enzyme inhibitors, penicillins, aminoglycosides, macrolides, tetracyclines, nitroimidazoles, fosfomycins, glycopeptides, lincosamides, macrodantin, sulfanilamides). These variables were analyzed to identify risk factors for CRKP infection.

Patients in the CRKP infection group were enrolled and divided into effective and non-effective groups to evaluate the impact of clinical outcomes. The definition of effective group with a positive outcome (cure or improvement) of CRKP infection was based on clinical (defeverscense, resolution or partial resolution of presenting symptoms, and signs of pneumoniae decline in suctioning requirements), radiological (decrease or disappearance of the findings on chest X-ray), and laboratory findings (improvement of arterial blood gasses and normalization of white blood cell count, C-reactive protein, and procalcitonin) (Michalopoulos et al., 2008). The therapeutic effect was studied by comparing the course of antibiotic treatment (first-line drugs: 3rd or 4th generation cephalosporins, carbapenems, quinolones, enzyme inhibitors; second-line drugs: tigecycline, fosfomycin, sulfanilamides) in CRKP-infected patients.

### Definitions

Carbapenem-resistant *Klebsiella pneumoniae* was defined according to the recommendation of the Center for Disease Control and Prevention^2^ (March 1, 2016) as KP strains resistant to at least one of the carbapenem agents including imipenem, ertapenem, and meropenem. The percentages of MDR, extensively drug-resistant (XDR) and pandrug-resistant (PDR) strains in CRKP were calculated according to the pre-specified criteria (Magiorakos et al., 2012).

The definitions of the site of infection and the responsible pathogen were based on the clinical symptoms and signs in individual patients, imaging findings, and on the isolation of a potential pathogen from evaluable clinical specimens. In addition, patients must have had fever >38°C with no other recognized cause, or abnormal white blood cell count [leukopenia (<4000 WBC/mm^3^) or leukocytosis (≥12,000 WBC/mm^3^)], and at least two of the following: new onset of purulent sputum or change in the sputum characteristics, increased respiratory secretions or increased suctioning requirements, new onset or worsening of a cough or dyspnea or tachypnea, rales or bronchial breath sounds, or worsening gas exchange (Falagas et al., 2005).

### Statistical Analysis

All the statistical analyses were performed by SPSS 18.0 (IBM Corp., Armonk, NY, United States). Continuous variables, summarized as means ± SD (*x̄* ± SD) or medians, were compared by Student’s *t*-test or the Mann–Whitney *U*-test. Qualitative variables were compared by χ^2^ or Fisher’s exact test. The univariate analysis was used for each variable. Two-sided *p*-values less than 0.05 were considered statistically significant. Variables with *p* < 0.05 were inserted into a binary logistic regression model for determining the potential independent risk factors associated with CRKP infection. Odds ratios (ORs), including their 95% confidence intervals (CIs), were calculated to evaluate the association between the factors and outcomes.

## Results

### Bacterial Isolates

In the current study, a total of 100 consecutive non-duplicate CRKP isolates were isolated from January 2014 to June 2015 from a tertiary teaching hospital in Shanghai. Isolates originated from different anatomical sites: sputum (*n* = 57, 57%), fluid (*n* = 12, 12%), blood (*n* = 12, 12%), urine (*n* = 11, 11%), bile (*n* = 3, 3%), cannula (*n* = 3, 3%), throat swab (*n* = 1, 1%), and pus (*n* = 1, 1%). CRKP isolates were obtained from patients admitted to the neurosurgery ward (*n* = 21, 21%), surgery ward (*n* = 16, 16%), geriatric ward (*n* = 15, 15%), surgery intensive care unit (SICU) ward (*n* = 14, 14%), emergency ward (*n* = 13, 13%), outpatients (*n* = 10, 10%), hematology ward (*n* = 5, 5%), nephrology ward (*n* = 2, 2%), endocrine ward (*n* = 1, 1%), liver transplant ward (*n* = 1, 1%), obstetrics and gynecology ward (*n* = 1, 1%), and urinary surgery ward (*n* = 1, 1%). Of the patients, 69 (69%) were males, 68 (68%) patients were >65 years-old (mean: 79.41 ± 8.77 years), and 32 (32%) patients were 18–65 years-old (mean: 50.50 ± 15.56 years).

### Antibiotic Susceptibility Test

The MIC range, MIC_50_ and MIC_90_ as well as the resistance rates of polymyxin B, tigecycline, ertapenem, meropenem, and imipenem among the 100 CRKP isolates are summarized in **Table 1**. The Kirby-Bauer disk diffusion test results showed that the resistance rates to the different drugs were MH 14%, SXT 16%, FOS 77%, AK 95%, ATM 99% and SCF 99%, and to the remaining (P, AMP, SAM, PIR, CFZ, CEC, CFM, CTX, CAZ, CIP, FEP, FOX) were 100%. The percentages of MDR, XDR and PDR were 50.0, 50.0, and 0%, respectively.

**Table 1:** Summary of MIC results among 100 CRKP isolates.

Antimicrobial agent	MIC range	MIC_50_	MIC_90_	R (%)	I (%)	S (%)
Polymyxin B	0.25–>128	1	8	25	0	75
Tigecycline	0.125–2	0.5	1	0	0	100
Ertapenem	16–>128	128	>128	100	0	0
Meropenem	2–>128	128	>128	99	1	0
Imipenem	4–128	64	128	100	0	0

*R, resistant; I, intermediate; S, sensitive.*

### Identification of Antibiotic Resistance Genes

All the isolates had positive results in the PCR with the primer pair detecting *bla*KPC-2, while only one strain was positive for metallo-carbapenemase *bla*IMP-1. For ESBLs, 96.0% (96/100), 1.0% (1/100), 84.0% (84/100), 75.0% (75/100), and 1% (1/100) of the isolates were detected as positive for *bla*CTX-M-9, *bla*CTX-M-15, *bla*SHV, and *bla*TEM, respectively. A total of 90% (90/100) isolates yielded PCR products with the primer pair detecting *qnr*B while only 8% (8/100) and 4% (4/100) were positive for *qnr*S and *qnr*A, respectively. Among all the investigated AmpC β-lactamase genes, only *bla*DHA was detected at a rate of 14.0% (14/100). None of the isolates were PCR-positive for *bla*CTX-M-1, *bla*CTX-M-25, *bla*OXA-48, *bla*VIM-1, *bla*VIM-2, *bla*IMP-2, *bla*NDM-1, *bla*EBC, *bla*CIT, *bla*FOX, *bla*ACC, or *bla*MOX. All details of the resistance gene profiles are listed in Supplementary Table 1.

### PFGE Analysis

**FIGURE 1:**
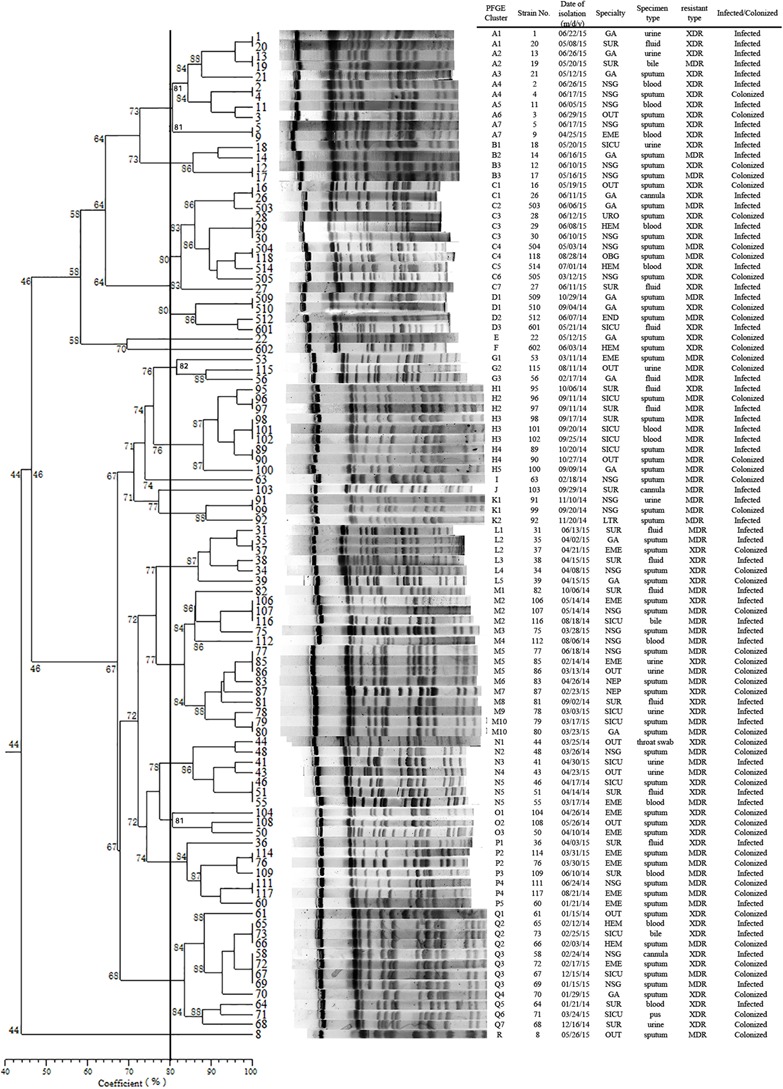
Dendrogram of the PFGE profiles of 100 CRKP isolates. The dendrogram was constructed using the UPGMA method. Dice coefficients (percentages) are listed in the scale under the dendrogram. NSG, neurosurgery ward; SUR, surgery ward; GA, geriatric ward; SICU, surgery intensive care unit; EME, emergency ward; OUT, outpatients; HEM, hematology ward; NEP, nephrology ward; END, endocrine ward; LTR, liver transplant ward; OBG, obstetrics and gynecology ward; URO, urinary surgery ward.

Pulsed-field gel electrophoresis patterns of the 100 *bla*KPC-2 producing CRKP isolates identified 18 PFGE types (clusters A–R) (**Figure 1**). No single dominant intra-hospital PFGE type was detected by using a cutoff of 80% similarity. Seven PFGE subtypes, including C3, H3, M2, M5, N5, Q2, and Q3, each contained at least three CRKP strains. Among them, all three of the CRKP strains in the H3 subtype were isolated from CRKP infection patients according to electronic medical records. In addition, according to epidemiological data such as the isolation date, department and specimen types, there were two CRKP detection peak periods (September, 2014–October, 2014 and April, 2015–June, 2015) involving three departments surgery, neurosurgery and geriatric ward. Details of PFGE clusters of CRKP strains isolated during those two detection peak periods were listed in **Table 2**.

**Table 2:** Pulsed-field gel electrophoresis (PFGE) clusters isolated during two CRKP detection peak periods.

Time period	Department	Num	PFGE type (n)
June 2014	SUR	6	H(3), M(2), Q(1)
April–June 2015	SUR	6	A(2), L(2), C(1), P(1)
	NSG	6	A(4), B(1), C(1)
	GA	9	A(3), C(2), L(2), B(1), E(1)

*SUR, surgery ward; NSG, neurosurgery ward; GA, geriatric ward; Num, the number of CRKP strains isolated during the time period.*

### Risk Factor Analysis

During the study period, 51 patients infected with CRKP and 51 controls (CSKP) were identified. In the CRKP infection groups, the respiratory tract (32/51, 62.7%) was the most common site infected with CRKP strains, followed by the bloodstream (5/51, 9.8%), urinary tract (5/51, 9.8%), surgical sites (5/51, 9.8%), central venous catheter (3/51, 5.9%), and digestive tract (1/51, 2.0%).

The comparison of the CRKP infection group to controls according to 39 clinical variables is listed in **Table 3**. Overall, the CRKP infection group was similar to the control group in baseline diseases except for the chronic pulmonary disease, which showed a relatively low incidence rate in the CRKP infection group (OR: 0.256, *p* = 0.038). Moreover, the CRKP infection cases were significantly more likely to have stayed in the ICU (OR: 3.684, *p* = 0.002), encountered increased exposure to invasive ventilation (OR: 2.973, *p* = 0.009), had blood transfusion (OR: 2.435, *p* = 0.028), parenteral nutrition (OR: 4.055, *p* = 0.004), undergone sputum suction (OR: 3.368, *p* = 0.006), or had high antibiotic exposure to carbapenems (OR: 2.889, *p* = 0.013), enzyme inhibitors (OR: 4.400, *p* = 0.021), nitroimidazoles (OR: 3.615, *p* = 0.029), and glycopeptides (OR: 4.531, *p* = 0.029). In addition, a large number of patients with CRKP infection had a medical history of hospital admission (OR: 2.670, *p* = 0.022) and exposure to antibiotics (OR 2.645, *p* = 0.03) 90 days before the outcome of interest.

**Table 3:** Risk factors for CRKP infection between the infected and control groups.

Variables	CRKP infection (*n* = 51)	Control group (*n* = 51)	Univariate analysis	
	*n* (%)	*n* (%)	OR (95% CI)	*p*-Value
**Demographics**				
Age (years, *x̄* ± SD)	69.84 ± 18.0	67.25 ± 20.1		0.457
Gender: male	39 (76.5)	35 (68.6)	1.486 (0.688-3.210)	0.375
**Baseline diseases**				
Hypertension	19 (37.3)	19 (37.3)	1.000 (0.499-2.004)	1.000
Diabetes mellitus	14 (27.5)	7 (13.7)	2.378 (1.033-5.479)	0.087
Heart disease	17 (33.3)	15 (29.4)	1.200 (0.583-2.469)	0.670
Malignancy	10 (19.6)	4 (7.8)	2.866 (1.055-7.786)	0.084
Chronic pulmonary disease	3 (5.9)	15 (19.6)	0.256 (0.072-0.908)	**0.038**
Liver disease	6 (11.8)	3 (5.9)	2.133 (0.652-6.982)	0.295
Hematologic disease	5 (9.8)	1 (2.0)	5.435 (1.016-29.061)	0.207
Nervous system disease	15 (29.4)	13 (25.5)	1.218 (0.576-2.576)	0.657
Nosocomial acquired infection	43 (84.3)	39 (76.5)	1.654 (0.684-3.997)	0.318
**Previous therapeutic process during hospital stay**				
Length of hospital stay prior to KP infection (days, median)	17	13		0.151
Immunosuppression	7 (13.7)	7 (13.7)	1.000 (0.377-2.656)	1.000
ICU admission	35 (68.6)	19 (37.3)	3.684 (1.803-7.530)	**0.002**
ICU stay (days, *x̄* ± SD)	24.6 ± 45.0	11.9 ± 31.5	-	0.084
Surgery	27 (52.9)	20 (39.2)	1.744 (0.885-3.437)	0.164
Invasive ventilation	27 (52.9)	14 (27.5)	2.973 (1.475-5.993)	**0.009**
Blood transfusion	28 (54.9)	17 (33.3)	2.435 (1.223-4.846)	**0.028**
Drainage tube	32 (62.7)	23 (45.1)	2.050 (1.030-4.083)	0.074
Parenteral nutrition	20 (39.2)	7 (13.7)	4.055 (1.829-8.992)	**0.004**
Nasogastric feeding tube	29 (56.7)	25 (29.0)	1.371 (0.697-2.697)	0.427
Sputum suction	23 (45.1)	10 (19.6)	3.368 (1.612-7.038)	**0.006**
Urinary catheter	37 (72.5)	31 (60.8)	1.705 (0.820-3.546)	0.208
Vessel catheter	44 (86.3)	37 (72.5)	2.378 (0.959-5.899)	0.087
**Medical history (90 days before KP infection)**				
Previous administration in hospital	23 (45.1)	12 (23.5)	2.670 (1.304-5.466)	**0.022**
Previous exposure to antibiotics	20 (39.2)	10 (19.6)	2.645 (1.256-5.571)	**0.030**
**Antibiotic exposure during hospital stay**				
Cephalosporins	37 (72.5)	33 (64.7)	1.442 (0.690-3.012)	0.393
Carbapenems	39 (76.5)	27 (52.9)	2.889 (1.358-6.145)	**0.013**
Quinolones	25 (49.0)	25 (49.0)	1.000 (0.510-1.959)	1.000
Enzyme inhibitors	11 (21.6)	3 (5.9)	4.400 (1.523-12.712)	**0.021**
Penicillins	7 (13.7)	8 (15.7)	0.855 (0.328-2.232)	0.780
Aminoglycosides	8 (15.7)	8 (15.7)	1.000 (0.397-2.520)	1.000
Macrolides	0 (0)	1 (2.0)	-	1.000
Tetracyclines	2 (3.9)	5 (9.8)	0.376 (0.079-1.782)	0.240
Nitroimidazoles	12 (23.5)	4 (7.8)	3.615 (1.371-9.531)	**0.029**
Fosfomycins	6 (11.8)	4 (7.8)	1.567 (0.513-4.785)	0.505
Glycopeptides	31 (60.8)	13 (25.5)	4.531 (2.212-9.282)	**0.000**
Sulfanilamides	2 (3.9)	1 (2.0)	2.041 (0.279-14.923)	1.000
Other lactams^∗^	5 (9.8)	1 (2.0)	5.435 (1.016-29.061)	0.092

*^∗^Other lactams: aztreonam, moxalactam. Bold values indicate significant *p* values < 0.05.*

The multivariate analyses of CRKP infection and control group models were based on the adjustment of the logistic regression model for 12 variables, wherein the risk factors differed significantly in the univariate analyses. As shown in **Table 4**, exposure to sputum suction was found to be the sole independent risk factor for CRKP infection.

**Table 4:** Multifactor logistics regression analysis of CRKP infection.

Risk factor	OR value	95% CI	*p*-Value
**Baseline diseases**			
Chronic Pulmonary Disease	0.333	0.055–2.012	0.231
**Previous therapeutic process during hospital stay**			
ICU admission	1.627	0.563–4.697	0.369
Invasive ventilation	1.363	0.406–4.579	0.616
Blood transfusion	1.790	0.534–5.994	0.345
Parenteral nutrition	2.181	0.623–7.626	0.222
Sputum suction	3.090	1.004–9.518	**0.049**
**Medical history (90 days before KP infection)**			
Previous administration in hospital	2.112	0.285–15.647	0.464
Previous exposure to antibiotics	1.817	0.235–14.057	0.567
**Antibiotic exposure during hospital stay**			
Carbapenems	1.681	0.542–5.216	0.368
Enzyme Inhibitors	1.651	0.312–8.738	0.555
Nitroimidazoles	1.318	0.265–6.545	0.736
Glycopeptides	2.416	0.838–6.963	0.102

*Bold values indicate significant p values < 0.05.*

### CRKP Infection Outcome Analysis

In 51 cases of CRKP infection, 28 patients (54.9%) were effective and 23 patients (45.1%) were non-effective, which were identified according to the preset criteria described in Section “Materials and Methods.” The usage and duration of various kinds of antibiotics were compared between the effective and non-effective treatment groups. As shown in **Table 5**, among all the antibiotics (cephalosporins, enzyme inhibitors, quinolones, carbapenems, fosfomycins, tigecycline, sulfanilamides), the treatment course (days) of carbapenems (*p* = 0.025) was a statistically significant factor.

**Table 5:** Duration of antibiotics treatment (*x̄* ± SD).

Antibiotics	Effective (*n* = 28)	Non-effective (*n* = 23)	*p*-Value
3rd, 4th generation cephalosporins, *n*	Nine cases	Eight cases	
3rd, 4th generation cephalosporins, *x̄* ± SD	3.78 ± 3.63	9.37 ± 12.96	0.138
Enzyme inhibitors, *n*	Five cases	Five cases	
Enzyme inhibitors, *x̄* ± SD	4.40 ± 2.19	4.60 ± 1.34	0.659
Tigecycline, *n*	Six cases	One case	
Tigecycline, *x̄* ± SD	9.00 ± 9.76	7.00 ± 0.00	0.857
Carbapenems, *n*	11 cases	13 cases	
Carbapenems, *x̄* ± SD	12.36 ± 9.11	6.23 ± 2.95	**0.002**
Quinolones, *n*	Nine cases	Five cases	
Quinolones, *x̄* ± SD	11.67 ± 9.04	6.40 ± 3.84	0.099
Sulfanilamides, *n*	Five cases	Three cases	
Sulfanilamides, *x̄* ± SD	12.36 ± 11.34	13.00 ± 11.7	0.933
Fosfomycins, *n*	Seven cases	One case	
Fosfomycins, *x̄* ± SD	14.14 ± 8.75	18.00 ± 0.00	0.694

*Bold values indicate significant p values < 0.05.*

## Discussion

Infections caused by carbapenem-resistant Enterobacteriaceae, especially by CRKP strains, are a pressing issue for public health. Although several attempts been made to control the spread of these infections at the local or national level, the rapid dissemination of CRKP has still posed an urgent threat globally (Girmenia et al., 2016; Lee et al., 2016). Because CRKP strains are either MDR, XDR or PDR, the available treatment protocols specific to the CRKP infected patients are limited. Although some combination therapy methods have been recommended for the treatment of CRKP infections, there is still a dearth of clinical evidence. Several studies have certified that CRKP, which is one of the most critical nosocomial pathogens, could increase the mortality rate, especially in those patients with severe diseases (Ducomble et al., 2015; Tumbarello et al., 2015). Therefore, our study combined the molecular epidemiology of clinical CRKP isolates and the study of risk factors for CRKP infection, discussed the epidemiological pattern, and evaluated different therapeutic effects in various treatments, to enable guidance in preventive measures and therapeutic strategies of CRKP infection in clinical applications.

In the current study, we used CRKP strains identified in patients between January 2013 and July 2015 and observed a distinct escalating trend during this period: CRKP-positive from 0.6% (4/656) in 2013 to 30.1% (142/472) in 2015. Focusing on the molecular detection and genetic characterization of CRKP, we found that the emergences of ESBL and carbapenemase genes were the primary resistance mechanisms. With respect to the ESBL genes, of which CTX-M type enzymes were reported as the most common type, 96% of CRKP isolates in our study harbored blaCTX-M-9, whereas only 1% contained the blaCTX-M-15 gene, which is by far the most prevalent CTX-M variant worldwide (Cantón et al., 2012). Much of this discrepancy is due to geographical area (Ranjbar et al., 2016), because blaCTX-M-9 and blaCTX-M-14, which belong to the CTX-M-9 group, were important CTX-M-positive strains in China (Cantón et al., 2012). On the other hand, among the carbapenemase genes, all the CRKP strains tested positive for *bla*KPC-2 while only one CRKP was positive for the metallo-carbapenemase gene *bla*IMP-1. The KPC enzyme was first reported in 1996 in a North Carolina (United States) hospital and was subsequently reported in several countries. Due to the emergence of diverse carbapenemase genes, KPC-2 is the most prevalent in China, with rare detection of metallo-carbapenemases, which was in agreement with our results. However, some other countries may have different dominant carbapenemases; for example, the United Kingdom is likely to have a mixed carbapenemase pattern with VIM and NDM, and NDM types followed by OXA-48-like types were prevalent in India (Munoz-Price et al., 2013). Moreover, the rate of positive detection of AmpC type β-lactamase was 14%, with only the DHA type being detected. DHA has been reported as the most frequent among AmpC β-lactamase-producing KP isolates in many studies (Shi et al., 2013; Liu and Liu, 2016), which was in line with our study. Therefore, DHA is speculated to be the most common type of AmpC β-lactamase in China.

We identified a total of 18 PFGE types (A-R) and 70 subtypes in 100 CRKP strains using PFGE analysis. Though no dominant intra-hospital PFGE type was identified using a cutoff of 80% similarity, two issues should be noted. Firstly, three PFGE H3 type CRKP strains were isolated from one surgical patient and from two patients in SICU later within 2 weeks. Although there is no direct epidemiological evidence showing the dissemination of H3 type CRKP strains from the surgical patient to the two SICU patients by medical records, the clustering distribution of PFGE H type clones in September to October 2014 in SUR and SICU departments indicated potential intra-hospital clonal transmission. Moreover, closely examining the two CRKP detection peak periods involving three departments (NSG, SUR, and GA), CRKP strains in high-risk ward concentrated in a certain period of time rather than being scattered throughout the study period. Therefore, epidemiological surveillance of drug-resistant isolates is vital and should be performed routinely, especially in high-risk departments.

Furthermore, our study shows that among the 100 CRKP strains, 51% isolates were from patients infected with CRKP while the remaining 49% were isolated from colonized patients. Unlike other studies, the respiratory tract was the most common infection site (32/51, 62.7%) followed by bloodstream (5/51, 9.8%) and urinary tract (5/51, 9.8%) while many other studies presented bacteremia (Falagas et al., 2007) and urinary tract (Teo et al., 2012) as the leading sites of infection. This may be correlated with the distribution of the specimen types sent to the clinical microbiology laboratory.

Univariate analyses revealed that the factors associated with the CRKP infection included 12 variables significantly different between the case (CRKP) and control (CSKP) groups. With respect to the baseline disease, the chronic pulmonary disease showed a low incidence rate in the case group compared to that in the control group with the OR value 0.256 (95% CI 0.072–0.908). Interestingly, if the pulmonary disease emerged as the underlying illness, it would be a protective factor for CRKP infection while if it occurred as a comorbidity disease, the opposite effect would occur (Lucena et al., 2014). In addition, ICU stays, various invasive manipulations, and prior medical history including admission to the hospital or exposure to antibiotics 90 days before the outcome of interest, were strongly correlated with CKPN infections, which has also been identified by other studies (Lucena et al., 2014; Hoxha et al., 2016; Lee et al., 2016). Regarding antibiotic exposure during hospital stays, the usage of carbapenems, enzyme inhibitors, nitroimidazoles, and glycopeptides was associated with CRKP infections. This prompted us to apply antibiotics according to the results of antimicrobial susceptibility testing instead of using broad spectrum antibiotics to prevent the increasing CRKP infections from causing in-hospital mortality.

Unlike other studies, only use of sputum suction (*p* = 0.006) was shown to be an independent risk factor in this study. Jiao et al. (2015) reported that exposure to glycopeptides, cefoperazone plus sulbactam, and tracheostomy were independent risk factors for CRKP infection/colonization. Falagas et al. (2007) conducted a case-control study and found that exposures to fluoroquinolones and antipseudomonal penicillin were independent risk factors for CRKP infections. Thus, nosocomial transmission may play a more critical role in the spread of CRKP than the selective pressure imposed by the usage of antibiotics in our hospital.

In addition, we further investigated the outcome of different treatment options for all the patients with infections caused by CRKP. Carbapenems play a major role in therapies treating infections caused by Enterobacteriaceae. However, the emergence of carbapenem-resistant Enterobacteriaceae has compromised the application of carbapenems in clinical practice. Because of this, we investigated the effectiveness of different antibiotic regimes in treating CRKP infection. In our study, only continual carbapenems usage was associated with greater treatment success. Although the antimicrobial susceptibility test showed *in vitro* resistance of bacteria to carbapenems, these antibiotics may be effective if the treatment period is prolonged. The results of this study may be partially influenced by the retrospective nature of data collected from the medical records and the treatment options, and the continuous utility period of the antibiotics may have been affected by medical insurance or patient’s financial capacity. However, consistent usage of carbapenems would be beneficial to CRKP**-**infected patients.

In summary, SICU, NSG, SUR, and GA were high-risk CRKP outbreak departments in our hospital, and epidemiological surveillance of resistance should be performed routinely. In the case-control study, sputum suction was an independent risk factor for CRKP infections. Prolonged utility period of carbapenems benefited patients infected with CRKP.

## Ethics Statement

This study was approved by The Institutional Review Board of the Renji Hospital, School of Medicine, Shanghai Jiaotong University, Shanghai, China. No consent was needed for this study.

## Author Contributions

BZ, YD, WS, ED, and DZ performed experiments. YY, YL, and YH assisted in data collection from the case and control groups. WS assisted in antimicrobial susceptibility testing. ML, BZ, YY, and WS conceived the study and analyzed the results. ML and BZ supervised the study and prepared the manuscript. All authors read and approved the final manuscript.

## Conflict of Interest Statement

The authors declare that the research was conducted in the absence of any commercial or financial relationships that could be construed as a potential conflict of interest.

## Footnotes

1 http://www.ncbi.nlm.nih.gov

2 www.cdc.gov/HAI/organisms/cre
