# Supplementary material for: Molecular Epidemiology and Risk Factors of Carbapenem-Resistant Klebsiella pneumoniae Infections in Eastern China
Source: Front Microbiol. 2017 Jun 13;8:1061. doi: 10.3389/fmicb.2017.01061 (PMC5468447; doi:10.3389/fmicb.2017.01061)
Supplement: Supplementary file 1 [file Table_1.DOCX]

Supplementary Material

Molecular epidemiology and risk factors of carbapenem-resistant Klebsiella pneumoniae infections in eastern region of China

Bing Zheng^#^, Yingxin Dai^#^, Yang Liu, Weiyang Shi, Erkuan Dai, Yichao Han, Dandan Zheng, Yuetian Yu, Min Li^*^

* Correspondence: Corresponding Author: [ruth_limin@126.com](mailto:ruth_limin@126.com)

## 1. Supplementary Table 1. Resistant gene profiles of 100 CRKP strains.

Supplementary Table 1. Resistant gene profiles of 100 CRKP strains.

| ID | *bla*KPC-2 | *bla*OXA-48 | *bla*VIM-1 | *bla*VIM-2 | *bla*IMP-1 | *bla*IMP-2 | *bla*NDM-1 | *bla*TEM | *bla*SHV | *bla*CTX-M-1 | *bla*CTX-M-9 | *bla*CTX-M-15 | *bla*CTX-M-25 | *bla*EBC | *bla*DHAM | *bla*CIT | *bla*FOX | *bla*ACC | *bla*MOX | *qnr*A | *qnr*B | *qnr*S |
| --- | --- | --- | --- | --- | --- | --- | --- | --- | --- | --- | --- | --- | --- | --- | --- | --- | --- | --- | --- | --- | --- | --- |
| 1 | + | - | - | - | - | - | - | + | + | - | + | - | - | - | - | - | - | - | - | - | + | - |
| 2 | + | - | - | - | - | - | - | + | + | - | + | - | - | - | - | - | - | - | - | - | + | - |
| 3 | + | - | - | - | - | - | - | + | + | - | + | - | - | - | - | - | - | - | - | - | + | - |
| 4 | + | - | - | - | - | - | - | + | + | - | + | - | - | - | - | - | - | - | - | - | + | - |
| 5 | + | - | - | - | - | - | - | + | + | - | + | - | - | - | - | - | - | - | - | - | + | - |
| 8 | + | - | - | - | - | - | - | + | + | - | + | - | - | - | + | - | - | - | - | - | + | - |
| 9 | + | - | - | - | - | - | - | + | + | - | + | - | - | - | - | - | - | - | - | - | + | - |
| 11 | + | - | - | - | - | - | - | + | + | - | + | - | - | - | - | - | - | - | - | - | + | - |
| 12 | + | - | - | - | - | - | - | - | + | - | + | - | - | - | - | - | - | - | - | + | + | - |
| 13 | + | - | - | - | - | - | - | + | + | - | + | - | - | - | + | - | - | - | - | - | + | - |
| 14 | + | - | - | - | - | - | - | + | + | - | - | - | - | - | + | - | - | - | - | - | + | - |
| 16 | + | - | - | - | - | - | - | + | + | - | + | - | - | - | + | - | - | - | - | - | + | - |
| 17 | + | - | - | - | - | - | - | - | + | - | + | - | - | - | - | - | - | - | - | - | + | - |
| 18 | + | - | - | - | - | - | - | - | + | - | + | - | - | - | + | - | - | - | - | - | + | - |
| 19 | + | - | - | - | - | - | - | - | + | - | + | - | - | - | + | - | - | - | - | - | + | - |
| 20 | + | - | - | - | - | - | - | + | + | - | + | - | - | - | + | - | - | - | - | - | + | - |
| 21 | + | - | - | - | - | - | - | + | - | - | + | - | - | - | - | - | - | - | - | - | + | - |
| 22 | + | - | - | - | - | - | - | + | + | - | + | - | - | - | - | - | - | - | - | - | - | - |
| 26 | + | - | - | - | - | - | - | + | + | - | + | - | - | - | - | - | - | - | - | - | + | - |
| 27 | + | - | - | - | - | - | - | + | - | - | + | - | - | - | - | - | - | - | - | - | + | + |
| 28 | + | - | - | - | - | - | - | + | + | - | + | - | - | - | - | - | - | - | - | - | + | + |
| 29 | + | - | - | - | - | - | - | - | + | - | + | - | - | - | - | - | - | - | - | - | + | - |
| 30 | + | - | - | - | - | - | - | + | + | - | + | - | - | - | - | - | - | - | - | - | + | - |
| 31 | + | - | - | - | - | - | - | + | + | - | + | - | - | - | - | - | - | - | - | - | + | - |
| 34 | + | - | - | - | - | - | - | + | + | - | + | - | - | - | - | - | - | - | - | - | + | - |
| 35 | + | - | - | - | - | - | - | + | + | - | + | - | - | - | - | - | - | - | - | - | + | - |
| 36 | + | - | - | - | - | - | - | - | + | - | + | - | - | - | - | - | - | - | - | - | + | - |
| 37 | + | - | - | - | - | - | - | - | - | - | + | - | - | - | - | - | - | - | - | - | + | - |
| 38 | + | - | - | - | - | - | - | + | - | - | + | - | - | - | - | - | - | - | - | - | + | - |
| 39 | + | - | - | - | - | - | - | + | + | - | + | - | - | - | - | - | - | - | - | - | + | - |
| 41 | + | - | - | - | - | - | - | + | + | - | + | - | - | - | - | - | - | - | - | - | + | + |
| 43 | + | - | - | - | - | - | - | + | + | - | + | - | - | - | - | - | - | - | - | - | + | - |
| 44 | + | - | - | - | - | - | - | + | + | - | - | - | - | - | - | - | - | - | - | - | + | + |
| 46 | + | - | - | - | - | - | - | - | - | - | + | - | - | - | - | - | - | - | - | - | + | - |
| 48 | + | - | - | - | - | - | - | + | + | - | + | - | - | - | - | - | - | - | - | - | + | - |
| 50 | + | - | - | - | - | - | - | + | + | - | + | - | - | - | + | - | - | - | - | + | - | - |
| 51 | + | - | - | - | - | - | - | + | + | - | - | + | - | - | - | - | - | - | - | - | + | - |
| 53 | + | - | - | - | - | - | - | - | - | - | + | - | - | - | - | - | - | - | - | - | + | - |
| 55 | + | - | - | - | - | - | - | + | + | - | + | - | - | - | - | - | - | - | - | - | + | - |
| 56 | + | - | - | - | - | - | - | + | - | - | + | - | - | - | - | - | - | - | - | - | + | - |
| 58 | + | - | - | - | - | - | - | + | + | - | + | - | - | - | - | - | - | - | - | - | - | - |
| 60 | + | - | - | - | - | - | - | - | + | - | + | - | - | - | - | - | - | - | - | - | + | - |
| 61 | + | - | - | - | - | - | - | + | - | - | + | - | - | - | - | - | - | - | - | - | + | + |
| 63 | + | - | - | - | - | - | - | + | + | - | + | - | - | - | - | - | - | - | - | + | - | - |
| 64 | + | - | - | - | - | - | - | + | + | - | + | - | - | - | - | - | - | - | - | - | - | - |
| 65 | + | - | - | - | - | - | - | + | - | - | + | - | - | - | - | - | - | - | - | - | + | - |
| 66 | + | - | - | - | - | - | - | + | + | - | + | - | - | - | - | - | - | - | - | - | - | - |
| 67 | + | - | - | - | - | - | - | + | + | - | + | - | - | - | - | - | - | - | - | - | + | - |
| 68 | + | - | - | - | - | - | - | - | + | - | + | - | - | - | - | - | - | - | - | - | + | - |
| 69 | + | - | - | - | - | - | - | - | + | - | + | - | - | - | - | - | - | - | - | - | + | - |
| 70 | + | - | - | - | - | - | - | - | + | - | + | - | - | - | - | - | - | - | - | - | + | - |
| 71 | + | - | - | - | - | - | - | + | + | - | + | - | - | - | - | - | - | - | - | - | - | - |
| 72 | + | - | - | - | - | - | - | - | + | - | + | - | - | - | - | - | - | - | - | - | + | - |
| 73 | + | - | - | - | - | - | - | + | + | - | + | - | - | - | - | - | - | - | - | - | + | - |
| 75 | + | - | - | - | - | - | - | + | + | - | + | - | - | - | - | - | - | - | - | - | + | - |
| 76 | + | - | - | - | - | - | - | - | + | - | + | - | - | - | - | - | - | - | - | - | - | - |
| 77 | + | - | - | - | - | - | - | - | - | - | + | - | - | - | - | - | - | - | - | - | + | - |
| 78 | + | - | - | - | - | - | - | + | - | - | + | - | - | - | - | - | - | - | - | - | + | - |
| 79 | + | - | - | - | - | - | - | + | - | - | + | - | - | - | - | - | - | - | - | - | + | - |
| 80 | + | - | - | - | - | - | - | + | + | - | + | - | - | - | - | - | - | - | - | - | + | - |
| 81 | + | - | - | - | - | - | - | + | + | - | + | - | - | - | - | - | - | - | - | - | + | - |
| 82 | + | - | - | - | - | - | - | - | + | - | + | - | - | - | + | - | - | - | - | - | + | - |
| 83 | + | - | - | - | + | - | - | + | + | - | + | - | - | - | + | - | - | - | - | - | + | + |
| 85 | + | - | - | - | - | - | - | + | - | - | + | - | - | - | + | - | - | - | - | - | + | - |
| 86 | + | - | - | - | - | - | - | - | - | - | + | - | - | - | + | - | - | - | - | - | + | - |
| 87 | + | - | - | - | - | - | - | + | + | - | + | - | - | - | + | - | - | - | - | - | + | - |
| 89 | + | - | - | - | - | - | - | + | + | - | + | - | - | - | - | - | - | - | - | - | + | - |
| 90 | + | - | - | - | - | - | - | + | + | - | + | - | - | - | - | - | - | - | - | - | + | - |
| 91 | + | - | - | - | - | - | - | + | + | - | + | - | - | - | - | - | - | - | - | - | - | - |
| 92 | + | - | - | - | - | - | - | + | + | - | + | - | - | - | - | - | - | - | - | - | + | - |
| 95 | + | - | - | - | - | - | - | + | + | - | + | - | - | - | - | - | - | - | - | - | + | - |
| 96 | + | - | - | - | - | - | - | + | + | - | + | - | - | - | - | - | - | - | - | - | - | - |
| 97 | + | - | - | - | - | - | - | + | + | - | + | - | - | - | - | - | - | - | - | - | + | - |
| 98 | + | - | - | - | - | - | - | - | + | - | + | - | - | - | - | - | - | - | - | - | + | - |
| 99 | + | - | - | - | - | - | - | - | + | - | + | - | - | - | - | - | - | - | - | - | + | - |
| 100 | + | - | - | - | - | - | - | + | + | - | + | - | - | - | - | - | - | - | - | - | + | - |
| 101 | + | - | - | - | - | - | - | + | + | - | + | - | - | - | - | - | - | - | - | - | + | - |
| 102 | + | - | - | - | - | - | - | + | + | - | + | - | - | - | - | - | - | - | - | - | + | - |
| 103 | + | - | - | - | - | - | - | + | + | - | + | - | - | - | - | - | - | - | - | - | + | - |
| 104 | + | - | - | - | - | - | - | - | + | - | + | - | - | - | - | - | - | - | - | - | + | - |
| 106 | + | - | - | - | - | - | - | + | - | - | + | - | - | - | - | - | - | - | - | - | + | - |
| 107 | + | - | - | - | - | - | - | + | + | - | + | - | - | - | - | - | - | - | - | - | + | - |
| 108 | + | - | - | - | - | - | - | - | + | - | + | - | - | - | - | - | - | - | - | - | + | - |
| 109 | + | - | - | - | - | - | - | - | + | - | + | - | - | - | - | - | - | - | - | - | + | - |
| 111 | + | - | - | - | - | - | - | - | + | - | + | - | - | - | - | - | - | - | - | - | + | - |
| 112 | + | - | - | - | - | - | - | + | + | - | + | - | - | - | - | - | - | - | - | - | + | - |
| 114 | + | - | - | - | - | - | - | - | + | - | + | - | - | - | - | - | - | - | - | - | + | - |
| 115 | + | - | - | - | - | - | - | + | + | - | + | - | - | - | - | - | - | - | - | - | + | + |
| 116 | + | - | - | - | - | - | - | + | + | - | + | - | - | - | - | - | - | - | - | - | + | - |
| 117 | + | - | - | - | - | - | - | + | + | - | + | - | - | - | - | - | - | - | - | - | + | - |
| 118 | + | - | - | - | - | - | - | + | - | - | + | - | - | - | - | - | - | - | - | - | + | - |
| 503 | + | - | - | - | - | - | - | + | + | - | + | - | - | - | - | - | - | - | - | - | + | - |
| 504 | + | - | - | - | - | - | - | + | + | - | + | - | - | - | + | - | - | - | - | + | + | - |
| 505 | + | - | - | - | - | - | - | + | + | - | + | - | - | - | - | - | - | - | - | - | + | - |
| 509 | + | - | - | - | - | - | - | + | + | - | + | - | - | - | - | - | - | - | - | - | + | - |
| 510 | + | - | - | - | - | - | - | + | + | - | + | - | - | - | - | - | - | - | - | - | + | - |
| 512 | + | - | - | - | - | - | - | + | + | - | + | - | - | - | - | - | - | - | - | - | + | - |
| 514 | + | - | - | - | - | - | - | + | + | - | + | - | - | - | - | - | - | - | - | - | + | - |
| 601 | + | - | - | - | - | - | - | + | + | - | + | - | - | - | - | - | - | - | - | - | + | + |
| 602 | + | - | - | - | - | - | - | + | + | - | - | - | - | - | - | - | - | - | - | - | + | - |
